# The Difference between Plasmon Excitations in Chemically Heterogeneous Gold and Silver Atomic Clusters

**DOI:** 10.3390/molecules29143300

**Published:** 2024-07-12

**Authors:** Fanjin Zeng, Lin Long, Shuyi Wang, Xiong Li, Shaohong Cai, Dongxiang Li

**Affiliations:** 1 College of Big Data and Information Engineering, Guizhou University, Guiyang 550025, China; fanjin_zeng139@163.com (F.Z.); lin_long64@126.com (L.L.); shuyiwang@gznc.edu.cn (S.W.); 2 College of Electronic and Information Engineering, Anshun University, Anshun 561000, China; 3 Guizhou Provincial Key Laboratory of Computational Nano-Material Science, Guizhou Education University, Guiyang 550018, China; 4 School of Science, East China University of Technology, Nanchang 330013, China; lix@ecut.edu.cn; 5 School of Information, Guizhou University of Finance and Economics, Guiyang 550025, China; 6 Department of Resources and Environment, Moutai Institute, Renhuai 564507, China

**Keywords:** time-dependent density functional theory, atomic clusters, plasmons, doping, electron transfer

## Abstract

Weak doping can broaden, shift, and quench plasmon peaks in nanoparticles, but the mechanistic intricacies of the diverse responses to doping remain unclear. In this study, we used the time-dependent density functional theory (TD-DFT) to compute the excitation properties of transition-metal Pd- or Pt-doped gold and silver atomic arrays and investigate the evolution characteristics and response mechanisms of their plasmon peaks. The results demonstrated that the Pd or Pt doping of the off-centered 10 × 2 atomic arrays broadened or shifted the plasmon peaks to varying degrees. In particular, for Pd-doped 10 × 2 Au atomic arrays, the broadened plasmon peak significantly blueshifted, whereas a slight red shift was observed for Pt-doped arrays. For the 10 × 2 Ag atomic arrays, Pd doping caused almost no shift in the plasmon peak, whereas Pt doping caused a substantial red shift in the broadened plasmon peak. The analysis revealed that the diversity in these doping responses was related to the energy positions of the d electrons in the gold and silver atomic clusters and the positions of the doping atomic orbitals in the energy bands. The introduction of doping atoms altered the symmetry and gap size of the occupied and unoccupied orbitals, so multiple modes of single-particle transitions were involved in the excitation. An electron transfer analysis indicated a close correlation between excitation energy and the electron transfer of doping atoms. Finally, the differences in the symmetrically centered 11 × 2 doped atomic array were discussed using electron transfer analysis to validate the reliability of this analytical method. These findings elucidate the microscopic mechanisms of the evolution of plasmon peaks in doped atomic clusters and provide new insights into the rational control and application of plasmons in low-dimensional nanostructures.

## 1. Introduction

Collective, coherent oscillations of conduction electrons in low-dimensional metal nanostructures under external excitation are called Localized Surface Plasmons (LSPs) [1,2,3]. For example, noble metal nanoparticles (such as Au and Ag) exhibit strong absorption peaks in the visible-to-infrared spectral range and are known as plasmon resonances [4]. These plasmon resonances can be tuned by altering the size [5], shape [6], spacing [7], composition [8], and dielectric environment [9] of the nanoparticles. Thus, they offer wide-ranging potential applications in optoelectronics [10], sensing [11], solar energy conversion [12], and biomedicine [13].

However, in many practical cases, noble metal nanoparticles are chemically heterogeneous [14,15], which introduces new opportunities and challenges to the application of noble metal nanoparticles. Even a small amount of impurities in noble metal nanoparticles will result in a complex evolution of their plasmonic properties, including the shift, broadening, and quenching of plasmon peaks. This challenge stimulates the enthusiasm for the doping modification of these low-dimensional nanostructures [16,17,18,19]. In experiments, Nilius et al. [20] first prepared gold and silver atomic chains on a NiAl(110) surface using STM and detected the presence of collective electron excitations. Wallis et al. [15] also used STM techniques to position transition-metal Pd atoms on specific sites of small gold chains on a NiAl(110) surface and revealed the existence of localized electron resonance states around the doped atoms. Experimentally, this finding proved the existence of plasmonic excitations in low-dimensional nanostructures composed of a few atoms and demonstrated that weak doping can modulate their plasmonic properties.

In theoretical calculations, Kummel et al. [21] computed sodium clusters using density functional theory and found plasmonic excitations even in sodium clusters composed of a small number of atoms. Subsequently, Lian et al. [22] investigated the excitation properties of small gold chains using the linear response time-dependent density functional theory (LR-TDDFT) and found that increased lengths of gold chains led to plasmonic excitations. Based on existing experimental and theoretical foundations, Nayyar et al. [17] studied the optical properties of transition-metal (Ni, Rh, Fe)-doped small gold chains using LR-TDDFT, revealed that doping broadened the plasmon peaks in small gold chains, and associated additional absorption peaks with a localized plasmon mode. Li et al. [23] also discussed the optical properties of Fe, Cu, Ni, and Rh atom-doped small silver chains using LR-TDDFT. They similarly observed varying degrees of broadening in the plasmon peaks of these small doped silver chains and suggested additional absorption peaks as a localized plasmon mode. However, when Chen et al. [18] theoretically calculated the optical properties of small gold chains doped with transition-metal nickel (Ni) atoms using real-time density functional theory (RT-TDDFT), they did not find the aforementioned localized plasmon mode. All plasmon peaks were influenced by impurities, and electronic oscillations of certain plasmon modes only occurred on half of the chain, which changed their excitation energies. Thus, there are divergent views on the mechanism of additional plasmon peak formation.

Recently, Conley et al. [19] investigated the properties of plasmon excitations in arrays of gold atoms doped with Cu and Pd atoms using RT-TDDFT. They followed the methodology in reference [24], analyzed the excited states through KS transition contributions, and revealed that the differences in the number of nodes between occupied and unoccupied electronic wavefunctions that participated in the transitions caused the variations in the transition energies. For example, the peak blue-shifted after the plasmon peaks broadened because the difference in the number of nodes between participating orbitals increased. However, the origin of differences in the number of nodes between occupied and unoccupied electronic wavefunctions that participate in excitations remains unresolved.

The differences in the plasmon excitation responses of weakly doped nanoparticles and the transfer mechanism of electrons during excitation comprise a micro-mechanism problem of plasmon evolution. Studying this problem can provide insights into the rational control and application of nanoparticle plasmons. Therefore, we used the time-dependent density functional theory (TD-DFT) to calculate the absorption spectra of 10 × 2 atomic arrays and their off-center doping with Pd or Pt. Then, we analyzed the electronic structure of atomic arrays, orbital transition contributions, and electron transfer. Finally, we confirmed the responsiveness of the centrally doped atoms using an electron transfer analysis method, demonstrating that this method can effectively explain the differences in doping responses. These findings elucidate the micro-mechanism of plasmon peak evolution in doped atomic clusters and provide new insights into the rational control and application of plasmons in low-dimensional nanostructures.

## 2. Results and Discussion

### 2.1. Discrepancies in Absorption Spectra Induced by Off-Center Doping

**Figure 1:**
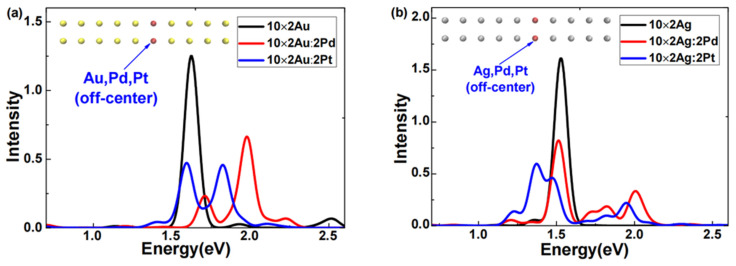
Absorption spectra of the (**a**) 10 × 2 Au:2X atomic array(X = Au, Pd, Pt), and (**b**) 10 × 2 Ag:2X atomic array(X = Ag, Pd, Pt).

The excited-state absorption spectra of 10 × 2 Au or 10 × 2 Ag atomic arrays doped with Pd or Pt off-center were obtained via LR-TDDFT calculations, as illustrated in Figure 1. Figure 1a shows that after the Pd or Pt doping, the absorption spectra of the 10 × 2 gold atomic array exhibited two distinct features. First, for either Pd or Pt doping, the plasmon peaks of the gold atomic array broadened into two clearly separated peaks. Second, for Pd-doped gold atomic arrays, both broadened plasmon peaks blueshifted, and the peak of higher intensity had a greater shift, which was remarkably consistent with the findings of Conley et al. [19] using RT-TDDFT analysis. Regarding the Pt doping, the 10 × 2 gold atomic array exhibited two plasmon peaks of comparable intensity; one peak slightly redshifted, the other significantly blueshifted, and they were situated between the two broadened plasmon peaks of the Pd-doped gold atomic arrays. In Figure 1b, after the Pd or Pt doping, the absorption spectra of the silver atomic array also displayed two distinct features. First, for either Pd or Pt doping, the plasmon peaks of the silver atomic array broadened. Second, for the Pd-doped silver atomic arrays, both broadened plasmon peaks slightly redshifted, whereas, for the Pt-doped silver atomic arrays, the higher-intensity peak significantly redshifted. This pronounced redshift of the plasmon peak of Pt-doped silver atomic arrays was particularly notable since previous doping response studies [17,19,23] typically observed blueshifts in broadened peaks.

Combining the characteristics of the absorption spectra in Figure 1a,b, we can conclude that for both Pd and Pt doping, the plasmon peaks of gold or silver atomic arrays broadened, which was consistent with the findings of Conley et al. [19] using RT-TDDFT analysis. However, there were substantial differences in the broadening and shifting of the plasmon peaks. For example, in Pt-doped silver atomic arrays, the higher-intensity plasmon peak significantly redshifted. This phenomenon clearly demonstrated that doping the same atomic species into different systems or doping different atoms into the same system results in markedly different effects. These sensitive differences in weak doping responses present new opportunities and challenges for the rational control of the optical properties and applications of nanoscale systems.

### 2.2. Analysis of the Electronic Structure of Atomic Arrays

To comprehend the aforementioned sensitivity to weak doping responses, we analyzed the electronic structure before and after the weak doping of atomic arrays.

**Figure 2:**
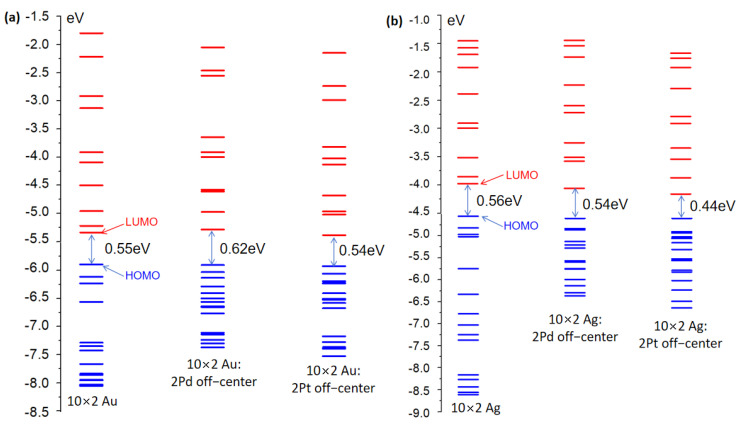
(**a**) Orbital energy diagrams of the 10 × 2 Au atomic arrays. (**b**) Orbital energy diagrams of the 10 × 2 Ag atomic arrays.

Initially, the electronic structures of the undoped 10 × 2 Au and 10 × 2 Ag atomic arrays were considered. The 10 × 2 Au and 10 × 2 Ag atomic arrays consisted of 20 Au and Ag atoms, respectively, as illustrated in Figure 1. Orbital energy diagrams of the 10 × 2 Au and 10 × 2 Ag atomic arrays were computed and included 15 occupied orbitals and 10 unoccupied orbitals, as shown in Figure 2. In the electronic structures of the gold and silver atomic arrays, molecular orbitals primarily constituted of 4d orbitals were fully occupied, while those composed of 5s and 5p orbitals were partially occupied. Figure 2a,b show that the 10 × 2 Au and 10 × 2 Ag atomic arrays had nearly identical band gaps (energy difference between HOMO and LUMO orbitals) (0.55 eV and 0.56 eV, respectively), but there were substantial disparities in the energy distributions of the 15 occupied and 10 unoccupied orbitals. The supplementary information in Tables S1 and S2 reveals that in the range of 15 occupied orbitals, the HOMO-4 of the 10 × 2 Au atomic array constituted a localized d-band, whereas the localized d-band appeared at HOMO-10 for the 10 × 2 Ag atomic array. The energy variations in the range of 15 occupied orbitals for the 10 × 2 Au and 10 × 2 Ag atomic arrays were 2.16 eV and 4.06 eV, respectively, with a difference of 1.19 eV. Hence, the d-band orbitals of the 10 × 2 Au atomic array occurred in a higher energy range than those of the 10 × 2 Ag atomic array. For the 10 unoccupied orbitals, the energy variations for the 10 × 2 Au and 10 × 2 Ag atomic arrays were 3.54 eV and 2.53 eV, respectively, with a difference of 1.01 eV. Hence, the 10 × 2 Au atomic array had a larger gap between unoccupied orbitals than the 10 × 2 Ag atomic array.

We considered the electronic structure of the 10 × 2 Au and 10 × 2 Ag atomic arrays doped with Pd and Pt atoms. For the 10 × 2 Pd-doped Au and 10 × 2 Ag atomic arrays, three distinct effects were observed. First, the change in the HOMO-LUMO gap differed: there was an increase of 0.07 eV for the 10 × 2 Au array and a decrease of 0.02 eV for the 10 × 2 Ag array upon Pd doping. Second, the gaps between unoccupied orbitals significantly decreased for 15 orbitals, whereas the gaps for the remaining 10 orbitals exhibited minor changes. There was also irregularity in the differences between pairs of unoccupied orbitals. Third, and most importantly, the supporting information in Tables S1–S3 shows that in the Pd-doped 10 × 2 Au array, the HOMO-3 orbital exhibited localized d-band characteristics, whereas HOMO-3 corresponded to the ∑_4_ orbital of the Pd dopant atom. Similarly, in the Pd-doped 10 × 2 Ag array, HOMO-5 corresponded to a hybridized orbital, HOMO-5 corresponded to the ∑_4_ orbital of the Pd dopant atom, and HOMO-6 exhibited localized d-band characteristics. Thus, Pd doping of the 10 × 2 Au and 10 × 2 Ag atomic arrays caused d-band orbitals to appear at higher energy levels. Additionally, orbitals that corresponded to the dopant Pd atom and hybridized orbitals with ambiguous nodal structures [24] emerged.

Next, the 10 × 2 Pt-doped Au and Ag atomic arrays were examined. Upon Pt doping, three distinct effects were observed on the 10 × 2 Au and 10 × 2 Ag atomic arrays. First, the HOMO-LUMO gap decreased for both arrays by 0.01 eV and 0.12 eV for the 10 × 2 Au and Ag arrays, respectively. The gap reduction in the 10 × 2 Ag array upon Pt doping was substantial, which caused a significant redshift in the plasmon excitations (this is further supported by specific data in the subsequent section). Second, the gaps between unoccupied orbitals significantly decreased, whereas the gaps for the remaining 10 orbitals exhibited similar changes to those upon Pd doping. Third, and also significantly, based on Table S1, in the Pt-doped 10 × 2 Au array, HOMO-1 corresponded to a hybridized orbital and HOMO-3 corresponded to the ∑_4_ orbital of the Pd dopant atom, but clear d-band characteristics were only evident at HOMO-12. In the Pt-doped 10 × 2 Ag array, HOMO-2 corresponded to a hybridized orbital, HOMO-4 corresponded to the ∑_4_ orbital of the Pd dopant atom, and no orbitals with localized d-band characteristics were observed in the range of the 15 unoccupied orbitals examined. Upon Pt doping, in both 10 × 2 Au and 10 × 2 Ag atomic arrays, the orbitals with pronounced localized d-band characteristics only appeared at relatively lower energy levels in the occupied orbitals.

In summary, contrasting the undoped 10 × 2 Au and 10 × 2 Ag atomic arrays revealed d-band orbitals at higher energy levels in the occupied orbitals of the 10 × 2 Au array, and there were larger gaps between unoccupied orbitals. Furthermore, comparing the Pd- and Pt-doped 10 × 2 Au and 10 × 2 Ag atomic arrays revealed the appearance of orbitals that corresponded to the dopant atoms in the occupied orbitals. However, the distinction was that in the Pd-doped 10 × 2 Au and 10 × 2 Ag atomic arrays, d-band orbitals emerged at relatively higher energy levels in the occupied orbitals, whereas, in the Pt-doped arrays, the orbitals with pronounced d-band characteristics appeared at relatively lower energy levels in the occupied orbitals. These findings and their specific impact on the absorption spectra were further corroborated and validated through a subsequent analysis of Kohn–Sham (K-S) orbital transition contributions.

### 2.3. Analysis of Kohn–Sham Orbital Transition Contributions

To comprehend the disparities in the absorption spectra of 10 × 2 atomic arrays perturbed by the off-central doping of Pd or Pt and substantiate and validate the findings of the aforementioned electronic structure analysis, Table 1 presents the transition contributions to the principal excitations that formed the plasmon peaks and the K-S orbital statistics.

From Table 1, in the undoped gold atomic array (10 × 2 Au), the primary excitations of the plasmon peak occurred from ground state S_0_ to excited state S_19_, with an excitation energy of 1.62 eV. These excitations predominantly involved four single-particle transitions with contributions exceeding 5%. In the transition with the highest contribution, the occupation orbital HOMO-3 transitioned to the unoccupied orbital LUMO+1, which accounted for 71.4% of the transition contribution. Following the methodology in reference [24], the variation in orbital nodal structures was examined. The occupation orbital HOMO-3 exhibited six longitudinal nodes and zero transverse nodes. The unoccupied orbital LUMO+1 exhibited seven longitudinal nodes and zero transverse nodes. Consequently, the difference in longitudinal nodal counts between these orbitals for the primary transition was 1. This result is completely consistent with the calculations of Conley et al. [19] using the RT-TDDFT method, where the difference in longitudinal nodal counts was consistently 1. The third-highest contribution from the occupation orbital HOMO-6 (d-band) represented a typical localized d-type orbital. In bulk materials, bands composed of sp-type orbitals are called conduction bands and those composed of d-type orbitals are called valence bands. Therefore, for the undoped gold atomic array (10 × 2 Au), the transition from ground state S_0_ to excited state S_19_ involved a minor proportion of interband single-particle transitions, which blueshifted the plasmon peak [8]. This result partially elucidates the relatively smaller excitation energy observed in the undoped silver atomic array (10 × 2 Ag) under identical unperturbed conditions.

It is noteworthy that the energy positions of d orbitals in Au and Ag differed. Yet, why was the excitation energy of the 10 × 2 Au pure gold atomic array and 10 × 2 Ag silver atomic array only 0.09 eV apart? It is known that plasmons are defined as collective oscillations of conduction electrons, and their frequency depends not only on the geometric constraints of sp valence electrons and the energy positions of d electrons but also on the hybridization between d and sp electrons [24]. In the pure system discussed here, 10 × 2 Au(Ag), the single particles primarily involved in collective excitations were sp valence electrons (as shown in Table 1: the weight of HOMO-6 with d-orbital characteristics participating in excitations in the 10 × 2 Au system was only 9.2%, whereas, in the 10 × 2 Ag system, single particles without d-orbital characteristics participated in excitations). Therefore, the energy of plasmons mainly depended on the system’s geometric size (geometric constraints), leading to very close absorption peaks of plasmons. This further illustrates that plasmons are collective oscillations of conduction electrons.

The present study investigated the Pd-doped gold atom array 10 × 2 Au:2Pd with two plasmon peaks. According to Table 1, the primary excitations that contributed to the low-energy peak involved transitions from ground state S_0_ to excited state S_51_, with an excitation energy of 1.71 eV. There were three main single-particle transitions with over 5% contribution. The transition with the highest contribution was from the occupied orbital HOMO-5 to the unoccupied orbital LUMO+3 and represented 54.8% of the total transition. The HOMO-5 orbital lacked transverse nodes and had six longitudinal nodes, whereas the LUMO+3 orbital lacked transverse nodes and had seven longitudinal nodes, which resulted in a longitudinal node difference of 1. Similarly, the primary excitations that contributed to the high-energy peak involved transitions from ground state S_0_ to excited state S_65_, with an excitation energy of 1.98 eV. There were four main single-particle transitions with over 5% contribution. The transition with the highest contribution was from the occupied orbital HOMO-2 to the unoccupied orbital LUMO+5 and represented 42.1% of the total transition. The HOMO-2 orbital lacked transverse nodes and had three longitudinal nodes, whereas the LUMO+5 orbital lacked transverse nodes and had eight longitudinal nodes, which resulted in a longitudinal node difference of 5. The correlation between longitudinal node difference and excitation energy supports the findings of [24].

Similarly, the Pt-doped gold atom array 10 × 2 Au:2Pt exhibited two plasmon peaks, as depicted in Table 1. The primary excitations that constituted the low-energy peak involved transitions from ground state S_0_ to excited state S_53_, with an excitation energy of 1.60 eV. There were five main single-particle transitions with over 5% contribution. The transition with the highest contribution was from the occupied orbital HOMO-6 to the unoccupied orbital LUMO+1 and represented 43.0% of the total transition contribution. The HOMO-6 orbital lacked transverse nodes and had six longitudinal nodes, whereas the LUMO+1 orbital lacked transverse nodes and had seven longitudinal nodes, so the longitudinal node difference was 1, which was similar to the node difference for the undoped 10 × 2 gold atom array. The excitation energy difference between low-energy and high-energy peaks was only 0.02 eV. For the high-energy peak, the longitudinal node difference was 5, so its excitation energy was 0.22 eV higher than that of the low-energy peak.

Therefore, the differences in the absorption spectra of the 10 × 2 gold atom arrays with off-central Pd or Pt doping were consistent with the findings of Conley et al. [19] In the 10 × 2 silver atom array with off-central Pd or Pt doping, the shifts of the two peaks were different from those observed in the Pd-doped 10 × 2 Au:2Pd gold atom array. In the case of 10 × 2 Ag:2Pd, the low-energy peak experienced almost no shift (excitation energy difference of only 0.02 eV). Furthermore, the high-energy peak primarily involved transitions from ground state S_0_ to excited state S_56_, with an excitation energy of 1.99 eV, which closely matched the observed high-energy peak of 1.98 eV for the Pd-doped 10 × 2 Au:2Pd.gold atom array. Similarly, Pt doping induced a significant redshift in the plasmon peak of the 10 × 2 Ag:2Pt silver atom array. The main excitations that caused the redshift involved transitions from ground state S_0_ to excited state S_35_, with an excitation energy of 1.37 eV, including four main transition pairs with over 5% contribution. However, an explanation that was only based on the difference in node structures failed to account for the observed redshift phenomenon. Therefore, an analysis from the electron transfer perspective was warranted.

In conclusion, the LR-TDDFT theoretical calculations for the Pd-doped 10 × 2 gold atom array exhibited a blueshift in the plasmon absorption peak, which is consistent with the findings of Conley et al. However, for the Pt-doped 10 × 2 silver atom array, the plasmon peak exhibited a redshift, which a purely nodal comparison of transition contributions could not reasonably explain. Hence, an analysis from the electron transfer perspective was necessary for a comprehensive understanding.

### 2.4. Analysis of the Inter-Fragment Electron Transfer between Fragments

By computing excited-state electronic distribution, one can gain insight into the characteristics of electron transfer. The following methods can be used: subtracting the excited-state density from the ground-state density, calculating the difference between excited-state atomic charges and ground-state atomic charges, etc. The amount of electron transfer from fragment *R* to *S* fragment during the electronic excitation process can be quantified as follows in Equation (1) [25]:

(1) $$Q_{R,S}=\Theta_{R,hole}\Theta_{S,ele}$$

$\Theta_{R,hole}$ is the proportion of excited electrons attributed to fragment *R* and $\Theta_{\begin{array}{l} S,ele \end{array}}$ is the proportion attributed to the destination fragment *S*. A greater proportion of electrons attributed to fragment *S* corresponds to a greater transfer from *R* to *S*. After defining the unidirectional electron transfer between fragments, one can define the net electron transfer between two fragments as the difference between transfers in both directions, as shown in Equation (2) [25]. Additionally, one can define the net electron change for a particular fragment as the sum of net electron transfers between that fragment and all other fragments, as shown in Equation (3) [25].

(2) $$p_{S\to R}=Q_{S,R}-Q_{R,S}$$

(3) $$\Delta p_{R}=\sum_{S\neq R}p_{S\to R}=\sum_{S\neq R}\left(Q_{S,R}-Q_{R,S}\right)$$

The diagonal elements of matric *Q* in the equation formally represent the “amount of electron transfer from a fragment to itself”. Physically, this value can be interpreted as the extent to which electrons are redistributed in the fragment due to excitation. To date, the literature has effectively used this method. For example, Zhao et al. [26] used electron transfer analysis to analyze excited-state electron distributions, which proved highly effective in understanding the photonic and photocatalytic properties of materials.

**Figure 3:**
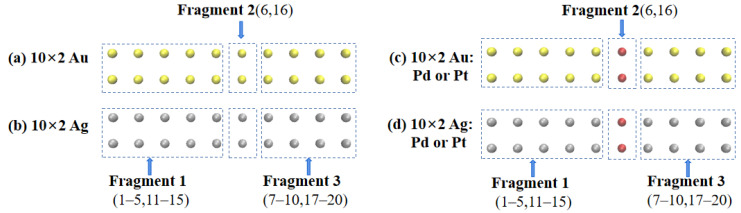
(**a**) Dividing a pure 10 × 2 Au atomic array into three fragments. (**b**) Dividing a pure 10 × 2 Ag atomic array into three fragments. (**c**) Dividing a 10 × 2 Au atomic array with off-center doped into three fragments. (**d**) Dividing a 10 × 2 Ag atomic array with off-center doped into three fragments.

**Figure 4:**
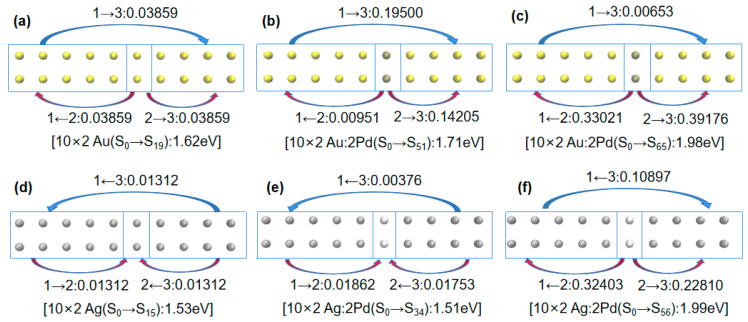

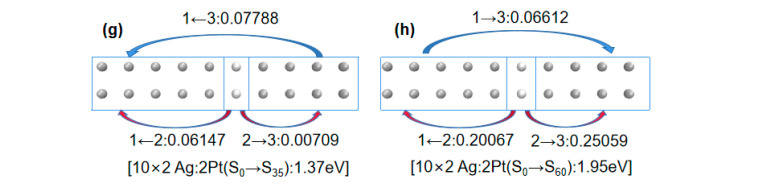
(**a**) Net electron transfer from the S_0_ state to the S_19_ state of the 10 × 2 Au atomic array. (**b**) Net electron transfer from the S_0_ state to the S_51_ state of the 10 × 2 Au:2Pd atomic array. (**c**) Net electron transfer from the S_0_ state to the S_65_ state of the 10 × 2 Au:2Pd atomic array. (**d**) Net electron transfer from the S_0_ state to the S_15_ state of the 10 × 2 Ag atomic array. (**e**) Net electron transfer from the S_0_ state to the S_34_ state of the 10 × 2 Ag:2Pd atomic array. (**f**) Net electron transfer from the S_0_ state to the S_56_ state of the 10 × 2 Ag:2Pd atomic array. (**g**) Net electron transfer from the S_0_ state to the S_35_ state of the 10 × 2 Ag:2Pt atomic array. (**h**) Net electron transfer from the S_0_ state to the S_60_ state of the 10 × 2 Ag:2Pt atomic array.(“→”or “←” represents the direction of electron transfer.).

In this study, the off-center doped atomic array was decomposed into three fragments: Fragment 1 consisted of gold or silver atoms with atomic numbers of 1–5 and 11–15; Fragment 3 consisted of gold or silver atoms with atomic numbers of 7–10 and 17–20; Fragment 2 consisted of gold or silver atoms with atomic numbers 6 and 16 when not doped and palladium or platinum atoms when doped, as shown in Figure 3. The net electron transfer among the three fragments for each system was computed and is summarized in Table 2. To visualize the net electron transfer among the fragments, Figure 4 presents the net electron transfer during the partial array excitations.

Combining Figure 3 and Figure 4 with Table 2 reveals that in the undoped scenario, the electron transfer between the gold atomic array (10 × 2 Au) and silver atomic array (10 × 2 Ag) among the three fragments was minimal. For example, in Figure 4a, transitioning from ground state S_0_ to excited state S_19_ in the gold atomic array (10 × 2 Au) involved a 0.03859 e transfer from Fragment 2 to Fragment 3. However, when palladium (Pd) was doped into the gold atomic array (10 × 2 Au:2Pd), as depicted in Figure 4b, the transition from ground state S_0_ to excited state S_51_ involved a 0.14205 e transfer from Fragment 2 to Fragment 3. In Figure 4c, the transitioning from ground state S_0_ to excited state S_65_ involved Fragment 2 transferring 0.33021 e and 0.39176 e to Fragment 1 and 3, respectively. Thus, when more electrons were transferred to the adjacent fragments due to the palladium doping, the excitation energy increased. Similar conclusions were drawn for platinum (Pt) doping in the gold atomic array (10 × 2 Au:2Pt), as indicated in Table 2.

Next, we examined Pd and Pt doping in a 10 × 2 silver atomic array. Combining Figure 1b and Table 2 reveals that in the 10 × 2 Ag:2Pd array, the absorption spectrum broadened into two plasmon peaks. Specifically, the plasmon peak from ground state S_0_ to excited state S_34_ was very close in position to that without doping. Comparing Figure 4d,e, they also had similar electron transfer characteristics; Fragment 1 and 3 transferred electrons to Fragment 2 and Fragment 3 transferred electrons to Fragment 1. However, there was a significant blueshift in the plasmon peak from ground state S_0_ to excited state S_56_ in Figure 4f, which indicated a much larger electron transfer from Fragment 2 (Pd atoms) to Fragment 1 and 3. Thus, the amount and direction of electron transfer in the same system determined the relative magnitude of excitation energy.

In particular, in the 10 × 2 Ag:2Pt array, a redshift occurred in the plasmon peak from ground state S_0_ to excited state S_35_. The aforementioned analysis, using the difference in electron wave function node numbers did not provide a reasonable explanation, so electron transfer analysis was used.

First, considering the electron transfer direction, a comparison of Figure 4e,g shows that Fragment 1 and 3 transferred electrons to Fragment 2 in the 10 × 2 Ag:2Pd array, whereas Fragment 2 transferred electrons to Fragment 1 and 3 in the 10 × 2 Ag:2Pt array. Thus, they had different electron distributions during excitation. For identical numbers of electrons, a further distribution corresponds to a smaller Coulomb interaction. Therefore, for the broadened low-energy peaks in the 10 × 2 Pd- and Pt-doped silver atomic arrays, the energy of the low-energy peak was lower for the Pt doping (1.37 eV) than for the Pd doping (1.51 eV).

Second, considering the electron transfer amount, a comparison of Figure 4g,h shows that in the 10 × 2 Ag:2Pt array, from ground state S_0_ to excited state S_35_, Fragment 2 transferred 0.06147 e and 0.00709 e to Fragment 1 and 3, respectively. However, from ground state S_0_ to excited state S_60_, Fragment 2 transferred 0.20067 e and 0.25059 e to Fragment 1 and 3, respectively. Clearly, similar to the patterns observed for the Pd- and Pt-doped gold atomic arrays, for the same system, a greater electron transfer transition corresponded to greater excitation energy.

**Figure 5:**
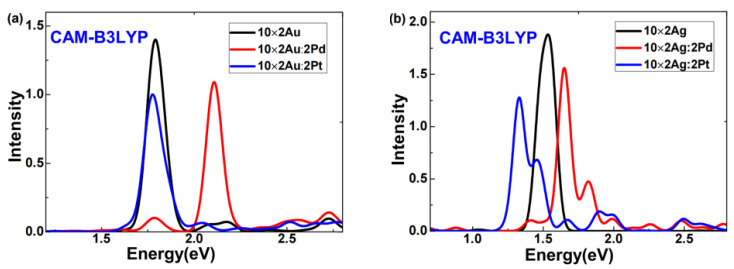
Absorption spectra of the (**a**) 10 × 2 Au:2X atomic array (X = Au, Pd, Pt), and (**b**) 10 × 2 Ag:2X atomic array (X = Ag, Pd, Pt). (employed the CAM-B3LYP functional).

Due to concerns that calculations with the GGA functional might overestimate electron transfer [27], we also employed the CAM-B3LYP functional to investigate pure and doped 10 × 2 Au (Ag) atomic arrays, aiming to assess whether B3PW91 functional calculations yielded unrealistic electron transfers. This study presents absorption spectra of pure and doped 10 × 2 Au (Ag) atomic arrays computed using the CAM-B3LYP functional combined with LANL2DZ, as shown in Figure 5. Comparing Figure 1 and Figure 5 reveals that while there were differences between spectra obtained using CAM-B3LYP and B3PW91 functionals, the trends in the blue shift and red shift of plasmon peaks after doping were consistent. Here, our focus is on examining whether B3PW91 functional calculations produced unrealistic electron transfers; hence, we do not discuss differences in plasmon peaks obtained from the two functionals.

Firstly, consider the doping of Pd and Pt in the gold atomic array. From Table 3, in the 10 × 2 Ag:2Pd array, the absorption spectrum showed two plasmonic peaks. Specifically, from the ground state s_0_ to the excited state S_37_, it exhibited similar electron transfer characteristics to the pure 10 × 2 gold atomic array from s_0_ to S_18_: minimal electron transfer between Fragments. However, from s_0_ to S_74_, Fragment 2 transferred electrons to Fragment 1 and 3, with values of 0.33021 e and 0.39176 e, respectively. This indicates that more electron transfer from Fragment 2 (Pd atoms) to Fragment 1 and 3 led to a more pronounced blue shift of the plasmon peak. In the 10 × 2 Ag:2Pt array, similar patterns in electron transfer and plasmon peak shifts were observed. This suggests that both functionals described the electron transfer and plasmon peak shifts equivalently.

Next, combining Table 2 and Table 3, we discuss whether B3PW91 functional calculations overestimated electron transfer. For the pure 10 × 2 Au atomic array, the B3PW91 functional calculated the electron transfer from Fragment 2 to Fragment 1 and 3 as 0.03859 e, while the CAM-B3LYP functional calculated it as 0.01160 e. Clearly, B3PW91 functional indeed overestimated electron transfer. However, for the pure 10 × 2 Ag atomic array, the B3PW91 functional calculated the electron transfer from Fragment 1 and 3 to Fragment 2 as 0.01312 e, whereas the CAM-B3LYP functional calculated it as 0.03942 e. This shows that the B3PW91 functional calculations underestimated electron transfer. Comparing other doping scenarios in Table 2 and Table 3 reveals that relative to the CAM-B3LYP functional, the B3PW91 functional can overestimate, underestimate, or approximate electron transfer, but the trend in electron transfer is generally consistent. Thus, the results of CAM-B3LYP functional calculations confirmed that using the B3PW91 functional to study the relationship between electron transfer and plasmon peak shifts is at least qualitatively feasible.

In summary, the broadening of plasmon peaks into different energy levels or the blue- or redshifts upon doping a certain system depends on the direction and magnitude of the electron transfer and is intricately linked to their competitive effects. Analysis based on the difference in the number of orbital nodes contributed by the transitions may sometimes prove ineffective in explaining these phenomena. However, introducing electron transfer analysis can offer another perspective to better understand the causes of excitation energy variations. CAM-B3LYP functional calculations also indicated that analyzing the relationship between electron transfer and plasmon peak shifts using the B3PW91 functional in this study was feasible.

### 2.5. Electron Transfer Analysis for Symmetric Center Doping

From the above discussion, the distinctiveness of asymmetric doping responses can be well explained by electron transfer analysis. To verify the universality of the electron transfer analysis in doping responses, we examined the differences in the plasmonic responses of 11 × 2 arrays of gold and silver atoms with center doping using electron transfer analysis.

**Figure 6:**
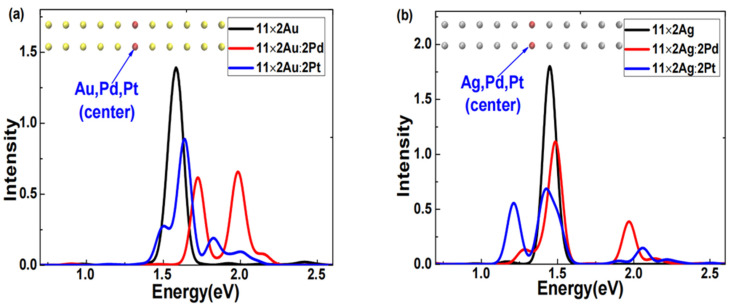
Absorption spectra of the (**a**) 11 × 2 Au:2X atomic array (X = Au, Pd, Pt), and (**b**) 11 × 2 Ag:2X atomic array (X = Ag, Pd, Pt).

First, using the same method, we obtained the excited states of symmetrically center-doped Pd or Pt in 11 × 2 gold and silver atom arrays. Figure 6 shows the absorption spectra broadened with Gaussian functions. Figure 6a shows two distinct features in the absorption spectra of the doped 11 × 2 gold atom array: the plasmon peak of the Pd-doped array broadened into two significantly blueshifted and stronger peaks; the plasmon peak of the Pt-doped array also broadened into multiple peaks, but only one was stronger and slightly blueshifted. Figure 6b shows two distinct features in the absorption spectra of the doped silver atom array: the plasmon peaks of both Pd- and Pt-doped silver atom arrays broadened into two clearly separated and stronger peaks; both plasmon peaks of the Pd-doped array exhibited varying degrees of blueshift, whereas one of the plasmon peaks of the Pt-doped array significantly redshifted. This result illustrates that the modulation effects produced by doping the same element into different arrays or doping different elements into the same array can significantly vary under center-symmetric doping conditions.

**Figure 7:**
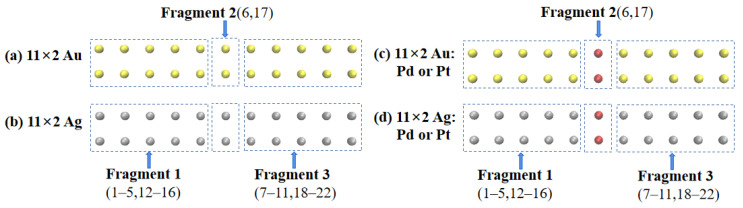
(**a)** Dividing a pure 11 × 2 Au atomic array into three fragments. (**b**) Dividing a pure 11 × 2 Ag atomic array into three fragments. (**c**) Dividing a 11 × 2 Au atomic array with symmetrically center-doped into three fragments. (**d**) Dividing a 11 × 2 Ag atomic array with symmetrically center-doped into three fragments.

**Figure 8:**
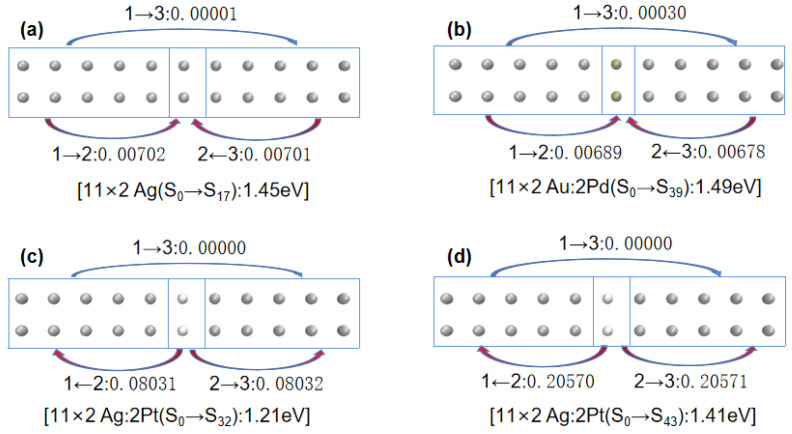
(**a**) Net electron transfer from the S_0_ state to the S_17_ state of the 11 × 2 Ag atomic array. (**b**) Net electron transfer from the S_0_ state to the S_39_ state of the 11 × 2 Au:2Pd atomic array. (**c**) Net electron transfer from the S_0_ state to the S_32_ state of the 11 × 2 Ag:2Pd atomic array. (**d**) Net electron transfer from the S_0_ state to the S_43_ state of the 11 × 2 Ag:2Pt atomic array.(“→”or “←” represents the direction of electron transfer.)

Next, to understand the differences in the excited-state absorption spectra of 11 × 2 gold and silver atom arrays that were symmetrically center-doped with Pd or Pt as described above, we decomposed the doped arrays into three Fragments: Fragment 1, consisting of gold or silver atoms with atomic numbers 1–5 and 12–16; Fragment 3, consisting of gold or silver atoms with atomic numbers 7–11 and 18–22; and Fragment 2, consisting of atoms with atomic numbers 6 and 17, as depicted in Figure 7. Table 3 shows the net electron transfer amounts between Fragments. To more intuitively understand the net electron transfer amounts among the three Fragments, Figure 8 illustrates selected array excitation electron net transfer models.

Combining Figure 7 and Figure 8, and Table 4, when Pd-doped 11 × 2 Au:2Pd atomic array transitioned from ground state S_0_ to excited state S_59_, the electron transfers from Fragment 1 and 3 to Fragment 2 were 0.02820 e and 0.02819 e, respectively. However, from ground state S_0_ to excited state S_76_, the electron transfers from Fragment 2 to Fragment 1 and 3 were 0.011257 e and 0.011261 e, respectively. When more electrons transferred from the doped Pd atoms to the two adjacent Fragments, the excitation energy increased. For the Pt-doped 11 × 2 Au:2Pt atomic array, there was minimal electron transfer between Fragments from ground state S_0_ to excited state S_62_, which was comparable to the undoped 11 × 2 Au atomic array. Hence, its excitation energy was very close to that of the undoped 11 × 2 Au atomic array (with a difference of only 0.04 eV).

For the 11 × 2 Ag:2Pd array, the electron transfer of the main excited states corresponded to the conclusions for the 11 × 2 Au:2Pd configuration, where more electrons transferring from the doped Pd atoms to the neighboring Fragments resulted in higher excitation energies. However, the situation was notably different for the 11 × 2 Ag:2Pt array. By transitioning from ground state S_0_ to excited state S_32_, Fragment 2 simultaneously transferred 0.08031 e and 0.08032 e to Fragment 1 and 3, respectively. Similarly, by transitioning from S_0_ to excited state S_43_, Fragment 2 transferred 0.20570 e and 0.20571 e to Fragment 1 and 3, respectively. More transferred electrons from the doped Pt atoms to the adjacent Fragments corresponded to greater excitation energy.

In particular, concerning the observed redshift phenomena in the last two main plasmon peaks after the Pt doping in the 11 × 2 Ag:2Pt array, similar interpretations could be made for the 10 × 2 Ag:2Pt array based on the direction and quantity of electron transfer. First, regarding the electron transfer direction, a comparison of Figure 8a,c reveals that for 11 × 2 Ag:2Pt, the low-energy peak transitions from the ground state (S_0_) to the excited state (S_32_) involved electron transfers toward the ends of the array; for undoped 11 × 2 Ag, the plasmon peaks transitioned from the ground state (S_0_) to the excited state (S_17_), with electron transfers toward the center of the array. When the electron distribution is relatively distant under equivalent electron numbers, the Coulomb interaction diminishes and results in relatively lower excitation energies. Second, regarding the electron transfer quantity, for the 11 × 2 Ag:2Pt array, the high-energy peak transitions from the ground state (S_0_) to the excited state (S_42_) involved electron transfers toward the ends of the array, with a substantial increase in transferred electron quantity, which led to relatively greater excitation energies.

Thus, the electron transfer analysis of symmetrically centered doping responses obtained similar conclusions to the off-center doping responses: in the same system, the quantity and direction of electron transfer between doped atoms and neighboring arrays determined the broadening direction, shifting direction, and magnitudes of the plasmon peaks in each excitation process.

## 3. Computational Details

In this study, we considered the possibility of utilizing scanning probes on specific material surfaces, with lattice steps transporting individual atoms to construct artificial quantum structures from the ground up, creating entirely new substances, materials, and devices. Therefore, this study adopted the model researched by Conley et al. [19], setting the atomic spacing to the experimental value of 2.89 Å [20]. Simultaneously, it assumed that the NiAl(110) surface did not affect the electronic properties of atomic clusters and proceeded with an idealized model of potential realistic clusters for the investigation. Regarding the question of how relaxation affects the spectrum of plasmon, in an earlier related work, it was found that absorption spectra are not very sensitive to changes in Au bond length in the range of 2.5 to 2.89 Å [17].

All calculations of the excited states were conducted using the Gaussian16 [28]. Specifically, the LR-TDDFT method was used with the B3PW91 functional [29,30] and LANL2DZ basis set [31,32,33] to simulate the excited states of the atomic arrays. The choice of the B3PW91 functional was based on two reasons. Firstly, this choice of functional and basis set allowed us to reproduce reasonably well the experimental results for the lowest excited states of the Au dimer [25,34]. Secondly, Conley et al., in the supplementary materials of references [19,24], compared the results of LR TD-DFT calculations performed at the B3PW91 theoretical level using the Gaussian03 with those obtained using the time-propagation TD-DFT method in the GPAW code package, showing consistent absorption spectrum characteristics [19]. Therefore, in this work, following references [19,24,25,34], LR TD-DFT calculations were conducted at the B3PW91 theoretical level. Both pure and doped 10 × 2 Au(Ag) atomic arrays were used with the CAM-B3LYP functional [35,36] and LANL2DZ basis set. Castro et al. reported in reference [37] that spin–orbit coupling significantly influences absorption spectrum resonances and oscillator strengths in nanowires, whereas, in more compact gold clusters, this spin–orbit effect is often quenched. Since this article primarily discusses the absorption spectra of atomic clusters, following the approach of references [19,38], only scalar relativistic effects were considered, without accounting for spin–orbit effects.

By preliminarily calculating the plasmon peak of a 10 × 2 gold atomic array, the LR-TDDFT method yielded a result of 1.62 eV, which was only 0.14 eV different from the result obtained by Conley et al. [19] using the RT-TDDFT method (1.48 eV). Regarding the discrepancies between the results of this study and the referenced papers, the slight shift in plasmon frequency was attributed to differences in the functionals [17]. Since this paper focuses on the differential modulation of plasmonic excitations in gold and silver atomic arrays induced by Pd or Pt doping, this marginal deviation did not substantively impact the research question.

Concerning the absorption spectra of the excited states, Gaussian functions such as that in Equation (4) were used for broadening [39].

(4) $$G(\omega )=\frac{1}{c\sqrt{2\pi }}e^{-\frac{{\left(\omega -\omega i\right)}^{2}}{2c^{2}}}(c=\frac{\mathrm{FWHM}}{2\sqrt{2\ln2}})$$

In Equation (1), *ω* is the spectral abscissa, and the full width at half maximum (FWHM) was set to 0.1 eV. The molecular orbital and electron transfer analyses in this study were conducted using the Multiwfn 3.8 (dev) code [39].

## 4. Conclusions

We used the LR-TDDFT method to study the evolution of plasmonic responses in Au and Ag atomic arrays doped with transition metals Pd or Pt and compared the microscopic mechanisms of the differences in doping responses. We reveal that doping a different system with the same type of atom or doping different atoms into the same system can produce drastically different effects. In particular, the plasmon peaks in 10 × 2 or 11 × 2 Ag atomic arrays substantially redshifted, regardless of whether the Pt doping was off-center or symmetrically centered. This effect is distinctly different from previous observations, where doping with atomic clusters typically led to a blueshift of plasmon peaks. A comparative analysis of the electronic structures showed that doping the Au and Ag atomic arrays with transition metals Pd or Pt introduced doped atom orbitals into the conduction band. However, there was a distinct difference: in 10 × 2 Au and 10 × 2 Ag arrays, Pd doping made the d-band orbitals appear at relatively higher occupied orbital energy levels than Pt doping. An analysis of the transitions contributed by K-S orbitals suggested that the doping altered the symmetry and gap between occupied and unoccupied orbitals, which involved multiple modes of single-particle transitions in the excitation process. High-energy excitations occurred due to the participation of transition orbitals, with greater differences in nodal count, whereas low-energy excitations occurred due to the differences in nodal count. The electron transfer analysis revealed that the excitation energies were closely linked to the electron transfers from the doped atoms. The amount and direction of electron transfer from the doping atoms to the remainder of the array on both sides determined the magnitude of the direction of the broadening and shift of the plasmon peaks. For either off-centered or symmetrically centered doping, if more electrons transferred from the doping atoms to the remaining parts of the array, the plasmon peaks had higher energies. The results of CAM-B3LYP functional calculations confirmed that it is feasible to analyze the relationship between electron transfer and plasmon peak shifts using the B3PW91 functional. In summary, this paper offers new insights into the rational control and application of plasmonic phenomena in low-dimensional nanosystems.

## Figures and Tables

**Table 1:** Kohn–Sham (K-S) transition contributions of the principal excitations induced by off-the central doping of Pd and Pt for the 10 × 2 atomic array.

Cluster	Peak	Excited State	Energy (eV)	Oscillator Strength	Transition (Delocalized Orbital Notion)	Weight (%)
10 × 2 Au	L	S_19_	1.62	3.80	HOMO-3(∑_7_)→LUMO+1(∑_8_) HOMO(∑_3_)→LUMO(∏_4_) HOMO-6(d-band)→LUMO(∏_4_) HOMO(∑_3_)←LUMO(∏_4_) HOMO-2(∏_1_)→LUMO+3(∏_6_)	71.4 15.7 9.2 −9.1 5.3
10 × 2 Au:2Pd	L1	S_51_	1.71	0.82	HOMO-5(∑_7_)→LUMO+3(∑_8_) HOMO-2(∑_4_)→LUMO+5(∑_9_) HOMO-6(hybridize)→LUMO+2(∏_3_)	54.8 14.5 8.4
	L2	S_65_	1.98	2.35	HOMO-2(∑_4_)→LUMO+5(∑_9_) HOMO-5(∑_7_)→LUMO+3(∑_8_) HOMO-1(∏_1_)→LUMO+6(∏_4_) HOMO-5(∑_7_)→LUMO+5(∑_9_)	42.1 12.7 9.2 7.4
10 × 2 Au:2Pt	L1	S_53_	1.60	1.54	HOMO-6(∑_7_)→LUMO+1(∑_8_) HOMO-7(hybridize)→LUMO+3(∏_3_) HOMO-4(hybridize)→LUMO+5(∑_9_) HOMO-14(d-band)→LUMO(∏_2_) HOMO-7(hybridize)→LUMO+4(∏_3_)	43.0 13.2 12.0 7.7 5.3
	L2	S_64_	1.82	1.34	HOMO-4(hybridize)→LUMO+5(∑_9_) HOMO-13(d-band)→LUMO+1(∑_8_) HOMO(∏_1_)→LUMO+6(∏_7_) HOMO-6(∑_7_)→LUMO+1(∑_8_) HOMO-7(hybridize)→LUMO+4(∏_3_)	19.6 14.6 13.6 12.6 11.7
10 × 2 Ag	L	S_15_	1.53	5.98	HOMO-3(∑_7_)→LUMO+1(∑_8_) HOMO(∏_3_)→LUMO(∏_4_) HOMO(∏_3_)←LUMO(∏_4_) HOMO-3∑_7_)←LUMO+1(∑_8_)	89.1 31.5 −16.0 −8.7
10 × 2 Ag:2Pd	L1	S_34_	1.51	3.04	HOMO-1(∑_7_)→LUMO+2(∑_8_) HOMO-3(∑_3_)→LUMO+2(∑_8_) HOMO-2(∏_1_)→LUMO+3(∏_3_) HOMO(∏_1_)→LUMO+1(∏_2_)	51.1 19.1 8.4 6.8
	L2	S_56_	1.99	0.79	HOMO-3(∑_3_)→LUMO+4(∑_9_) HOMO-12(∑_5_)→LUMO+2(∑_8_)	64.7 13.3
10 × 2 Ag:2Pt	L1	S_35_	1.37	2.08	HOMO-1(∑_7_)→LUMO+1(∑_8_) HOMO-3(∏_1_)→LUMO+3(∏_3_) HOMO-9(hybridize)→LUMO(∏_2_) HOMO(∏_1_)→LUMO+2(∏_2_)	37.7 28.6 14.1 5.8
	L2	S_60_	1.95	0.79	HOMO-9(hybridize)→LUMO+3(∏_3_) HOMO-12(∑_5_)→LUMO+1(∑_8_) HOMO-9(hybridize)→LUMO+2(∏_2_) HOMO-13(hybridize)→LUMO+1(∑_8_)	35.9 23.8 17.0 5.3

**Table 2:** Net electron transfer between fragments in the off-center doped atomic array for the 10 × 2 configuration (employed the B3PW91 functional).

Cluster	Excited State	Energy (eV)	Charge Transfer (e) (Fragment 1 to 2)	Charge Transfer (e) (Fragment 1 to 3)	Charge Transfer (e) (Fragments 2 to 3)
10 × 2Au	S_19_	1.62	1→2: −0.03859	1→3: 0.03859	2→3: 0.03859
10 × 2 Au:2Pd	S_51_	1.71	1→2: −0.00951	1→3: 0.19500	2→3: 0.14205
	S_65_	1.98	1→2: −0.33021	1→3: 0.00653	2→3: 0.39176
10 × 2 Au:2Pt	S_53_	1.60	1→2: −0.04857	1→3: 0.02562	2→3: 0.04520
	S_64_	1.82	1→2: −0.13906	1→3: −0.09329	2→3: 0.07033
10 × 2 Ag	S_15_	1.53	1→2: 0.01312	1→3: −0.01312	2→3: −0.01312
10 × 2 Ag:2Pd	S_34_	1.51	1→2: 0.01862	1→3: −0.00376	2→3: −0.01753
	S_56_	1.99	1→2: −0.32403	1→3: −0.10897	2→3: 0.22810
10 × 2 Ag:2Pt	S_35_	1.37	1→2: −0.06147	1→3: −0.07788	2→3: 0.00709
	S_60_	1.95	1→2: −0.20067	1→3: 0.06612	2→3: 0.25059

**Table 3:** Net electron transfer between fragments in the off-center doped atomic array for the 10 × 2 configuration (employed the CAM-B3LYP functional).

Cluster	Excited State	Energy (eV)	Charge Transfer (e) (Fragment 1 to 2)	Charge Transfer (e) (Fragment 1 to 3)	Charge Transfer (e) (Fragments 2 to 3)
10 × 2 Au	S_18_	1.78	1→2: −0.01160	1→3: 0.01160	2→3: 0.01160
10 × 2 Au:2Pd	S_37_	2.11	1→2: 0.07577	1→3: 0.03352	2→3: −0.06085
	S_74_	2.73	1→2: −0.33021	1→3: 0.00653	2→3: 0.39176
10 × 2 Au:2Pt	S_33_	1.77	1→2: −0.04919	1→3: 0.01830	2→3: 0.06822
	S_61_	2.52	1→2: −0.17154	1→3: −0.01313	2→3: 0.12402
10 × 2 Ag	S_11_	1.56	1→2: 0.03942	1→3: −0.03942	2→3: −0.03942
10 × 2 Ag:2Pd	S_19_	1.65	1→2: 0.06478	1→3: 0.16338	2→3: 0.02295
	S_51_	2.63	1→2: −0.15068	1→3: 0.00945	2→3: 0.12012
10 × 2 Ag:2Pt	S_18_	1.33	1→2: −0.10318	1→3: 0.03562	2→3: 0.07905
	S_37_	1.99	1→2: −0.31590	1→3: −0.12478	2→3: 0.20104

**Table 4:** Net electron transfer between doped Fragments in the symmetrically center-doped 11 × 2 atomic array.

Cluster	Excited State	Energy (eV)	Charge Transfer (e) (Fragment 1 to 2)	Charge Transfer (e) (Fragment 1 to 3)	Charge Transfer (e) (Fragments 2 to 3)
11 × 2 Au	S_23_	1.60	1→2: 0.05130	1→3: 0.00004	2→3: −0.05129
11 × 2 Au:2Pd	S_59_	1.72	1→2: 0.02820	1→3: 0.00002	2→3: −0.02819
	S_76_	1.98	1→2: −0.11257	1→3: 0.00014	2→3: 0.11261
11 × 2 Au:2Pt	S_62_	1.64	1→2: −0.06411	1→3: 0.00001	2→3: 0.06411
	S_68_	1.82	1→2: −0.39533	1→3: −0.00000	2→3: 0.39533
11 × 2 Ag	S_17_	1.45	1→2: 0.00702	1→3: 0.00001	2→3: −0.00701
11 × 2 Ag:2Pd	S_39_	1.49	1→2: 0.00689	1→3: 0.00030	2→3: −0.00678
	S_63_	1.97	1→2: −0.28836	1→3: 0.00012	2→3: 0.28835
11 × 2 Ag:2Pt	S_32_	1.21	1→2: −0.08031	1→3: 0.00000	2→3: 0.08032
	S_43_	1.41	1→2: −0.20570	1→3: 0.00000	2→3: 0.20571

## Data Availability

Data are contained within the article and Supplementary Materials.

## Supplementary Materials

The following supporting information can be downloaded at: https://www.mdpi.com/article/10.3390/molecules29143300/s1, Table S1: The delocalized orbital notion of the gold clusters; Table S2: The Kohn-Sham orbital graphics of the gold clusters (10 × 2 Au); Table S3: The Kohn-Sham orbital graphics of the gold clusters (10 × 2 Au:2Pd).
