# Supplementary material for: The Difference between Plasmon Excitations in Chemically Heterogeneous Gold and Silver Atomic Clusters
Source: Molecules. 2024 Jul 12;29(14):3300. doi: 10.3390/molecules29143300 (PMC11279591; doi:10.3390/molecules29143300)
Supplement: Supplementary file 1 [file molecules-29-03300-s001.zip › molecules-3041959-supplementary.pdf]

Support information

The Difference between Plasmon Excitations in Chemically Heterogeneous Gold and Silver Atomic Clusters

Fanjin Zeng [1,2], Lin Long [1], Shuyi Wang [1,3], Xiong Li [4], Shaohong Cai [1,5,6,*] and Dongxiang Li [1,2,*]

[1] College of Big Data and Information Engineering, Guizhou University, Guiyang 550025, China; FanJin_Zeng139@163.com (F.Z.); lin_long64@126.com (L.L.); shuyiwang@gznc.edu.cn (S.W.)
[2] College of Electronic and Information Engineering, Anshun University, Anshun 561000, China
[3] Guizhou Provincial Key Laboratory of Computational Nano-Material Science, Guizhou Education University, Guiyang 550018, China
[4] School of Science, East China University of Technology, Nanchang 330013, China; lix@ecut.edu.cn
[5] School of Information, Guizhou University of Finance and Economics, Guiyang 550025, China
[6] Department of Resources and Environment, Moutai Institute, Renhuai 564507, China
* Correspondence: shcai@gzu.edu.cn (S.C.); ldx0601@163.com (D.L.)

Table S1. The delocalized orbital notion of the gold clusters

**Kohn-Sham orbital**	**10×2 Au**		**10×2 Au:2Pd**		**10×2 Au:2Pt**	
	**Enegry (eV)**	**Delocalized orbital notion**	**Enegry(eV)**	**Delocalized orbital notion**	**Enegry(eV)**	**Delocalized orbital notion**
LUMO+9	-1.80	$\prod_1$	-2.05	$\prod_9$	-2.15	$\prod_9$
LUMO+8	-2.21	$\prod_9$	-2.46	$\sum_{10}$	-2.74	$\sum_{10}$
LUMO+7	-2.92	$\sum_{10}$	-2.55	$\prod_8$	-2.99	$\prod_8$
LUMO+6	-3.13	$\prod_8$	-3.64	$\prod_4$	-3.82	$\prod_7$
LUMO+5	-3.91	$\prod_7$	-3.91	$\sum_9$	-4.02	$\sum_9$
LUMO+4	-4.09	$\sum_9$	-4.00	$\prod_3$	-4.13	$\prod_3$
LUMO+3	-4.50	$\prod_6$	-4.58	$\sum_8$	-4.68	$\prod_3$
LUMO+2	-4.96	$\prod_5$	-4.61	$\prod_3$	-4.96	$\prod_2$
LUMO+1	-5.22	$\sum_8$	-4.98	$\prod_2$	-5.02	$\sum_8$
LUMO	-5.34	$\prod_4$	-5.29	$\prod_2$	-5.39	$\prod_2$
HOMO	-5.89	$\sum_3$	-5.91	$\prod_1$	-5.93	$\prod_1$
HOMO-1	-6.11	$\prod_2$	-6.03	$\prod_1$	-6.06	hybridi
HOMO-2	-6.23	$\prod_1$	-6.13	$\sum_4$	-6.19	$\prod_1$
HOMO-3	-6.56	$\sum_6$	-6.29	d-band	-6.23	$\sum_{4\text{-}Pt}$
HOMO-4	-7.29	d-band	-6.40	$\sum_{4\text{-}Pd}$	-6.23	hybridize
HOMO-5	-7.35	$\sum_6$	-6.50	$\sum_7$	-6.41	hybridize
HOMO-6	-7.43	d-band	-6.56	hybridize	-6.51	$\sum_7$
HOMO-7	-7.67	d-band	-6.64	hybridize	-6.52	hybridize
HOMO-8	-7.84	$\sum_5$	-6.65	hybridize	-6.58	hybridize
HOMO-9	-7.86	d-band	-6.76	hybridize	-6.67	hybridize
HOMO-10	-7.95	d-band	-7.11	$\sum_{3\text{-}Pd}$	-7.17	$\sum_{3\text{-}Pt}$
HOMO-11	-7.95	d-band	-7.14	$\sum_6$	-7.28	$\sum_6$
HOMO-12	-8.03	d-band	-7.24	hybridize	-7.37	d-band
HOMO-13	-8.04	d-band	-7.30	d-band	-7.40	d-band

HOMO-14	-8.05	d-band	-7.37	d-band	-7.53	d-band

Table S2. The Kohn-Sham orbital graphics of the gold clusters(10×2 Au)

HOMO−14	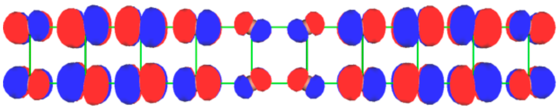
HOMO−13	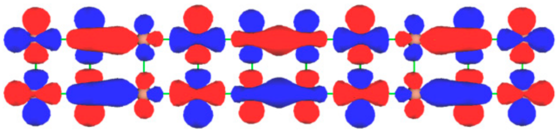
HOMO−12	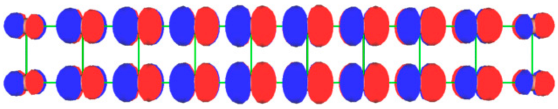
HOMO−11	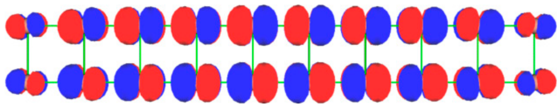
HOMO−10	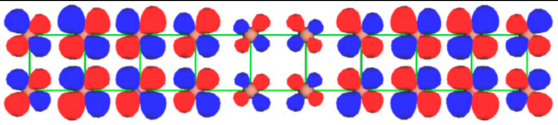
HOMO−9	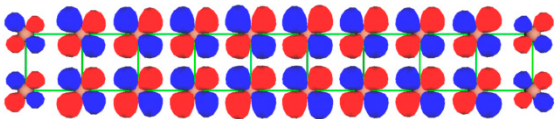
HOMO−8	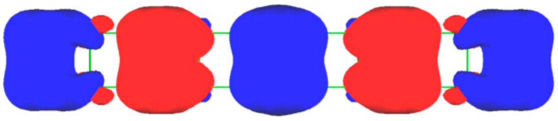
HOMO−7	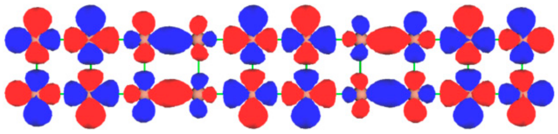
HOMO−6	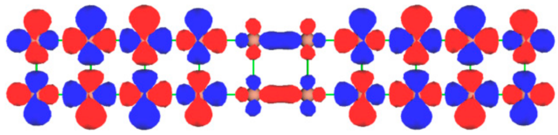
HOMO−5	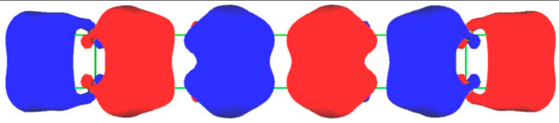
HOMO−4	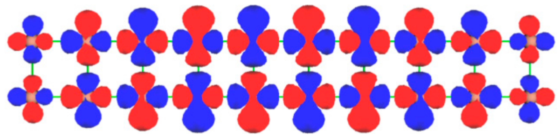

HOMO-3	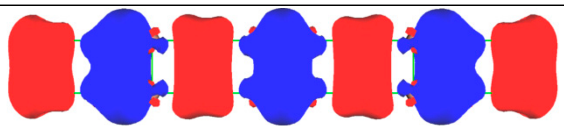
HOMO-2	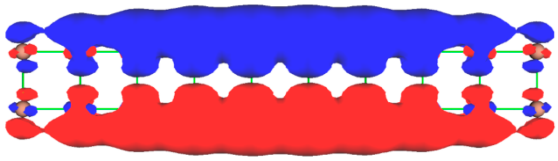
HOMO-1	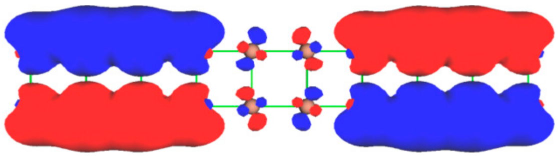
HOMO-0	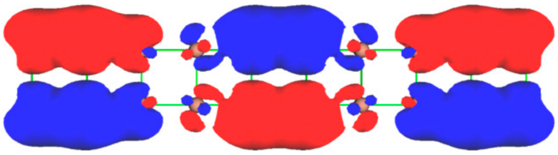
LUMO-0	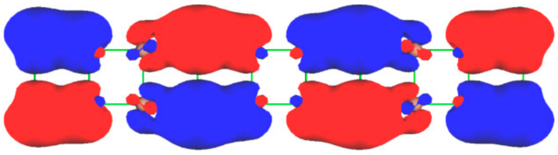
LUMO+1	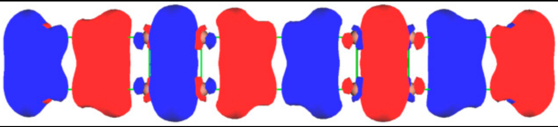
LUMO+2	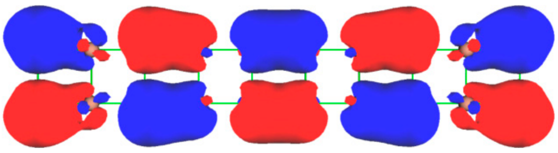
LUMO+3	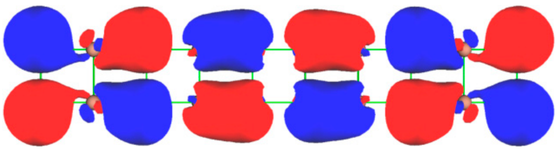
LUMO+4	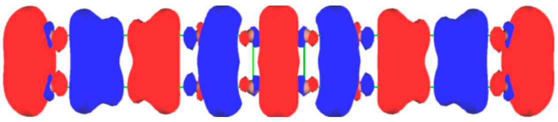
LUMO+5	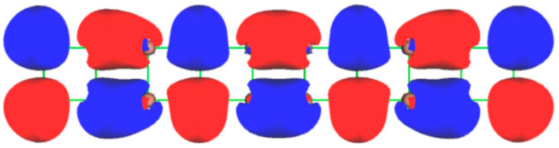
LUMO+6	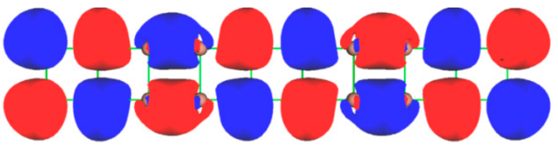
LUMO+7	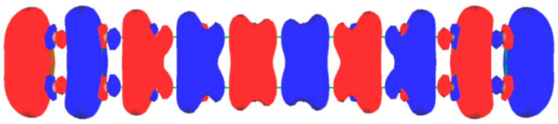

LUMO+8	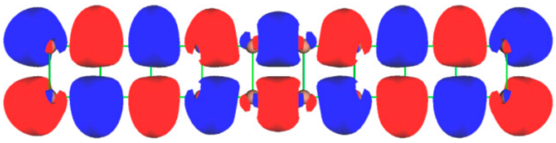
LUMO+9	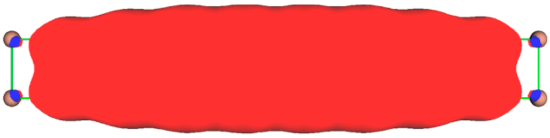

Table S3. The Kohn-Sham orbital graphics of the gold clusters(10×2 Au:2Pd)

HOMO−14	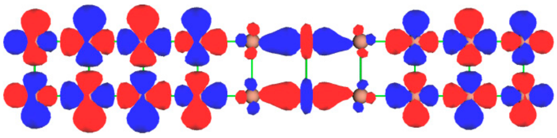
HOMO−13	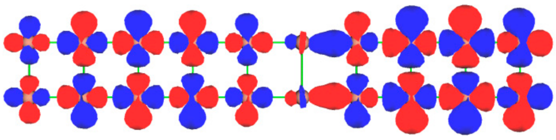
HOMO−12	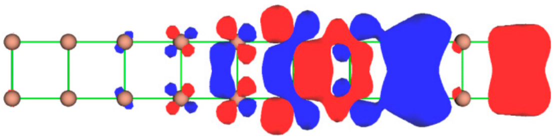
HOMO−11	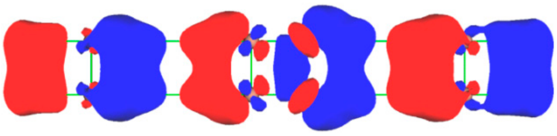
HOMO−10	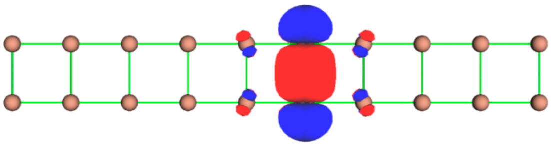
HOMO−9	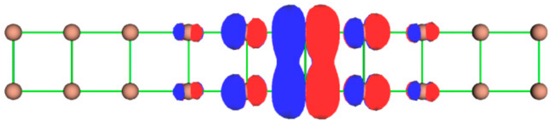
HOMO−8	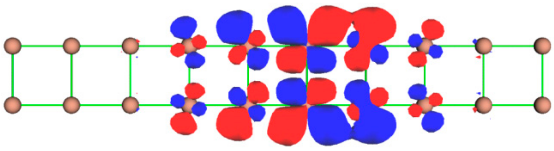
HOMO−7	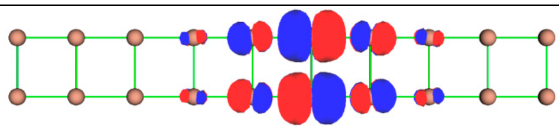

HOMO−6	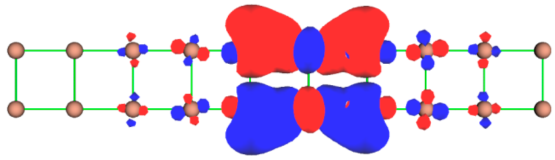
HOMO−5	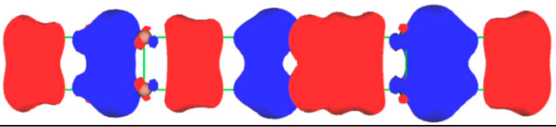
HOMO−4	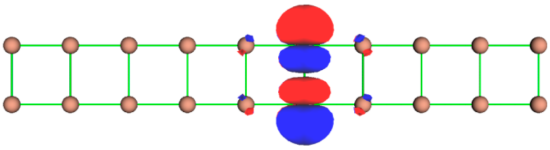
HOMO−3	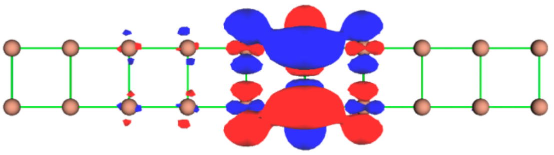
HOMO−2	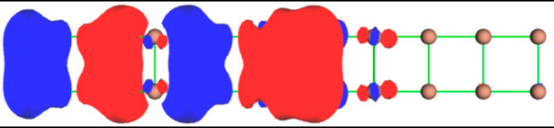
HOMO−1	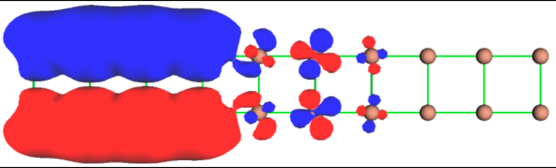
HOMO−0	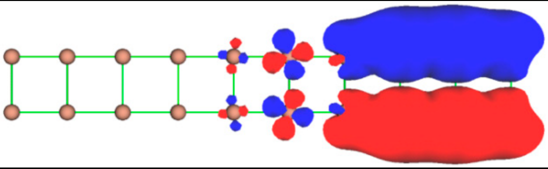
LUMO−0	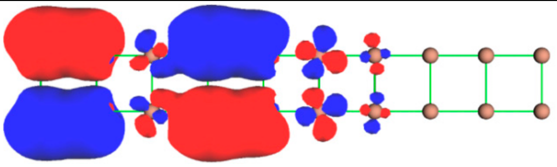
LUMO+1	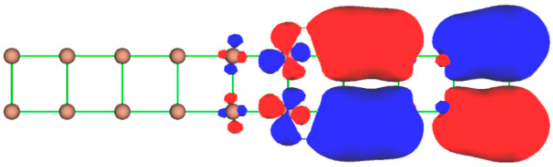
LUMO+2	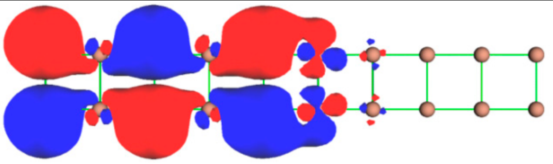
LUMO+3	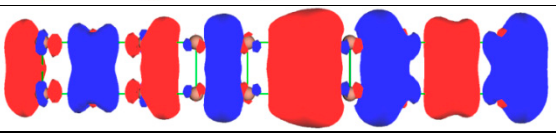

LUMO+4	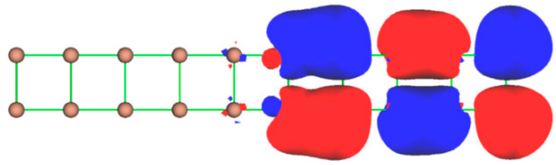
LUMO+5	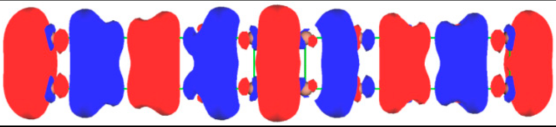
LUMO+6	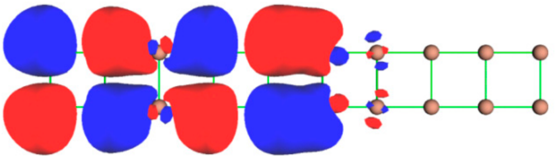
LUMO+7	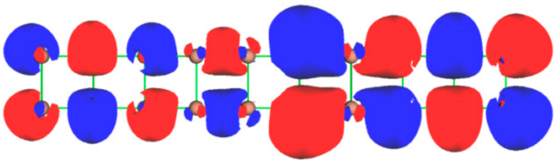
LUMO+8	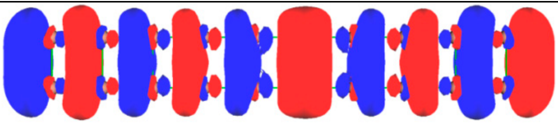
LUMO+9	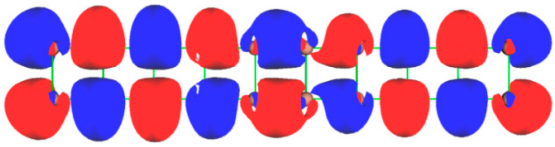
